# Editorial: Molecular and network mechanisms underlying stress resilience

**DOI:** 10.3389/fnmol.2024.1404375

**Published:** 2024-04-15

**Authors:** Frederick L. Hitti, Brian F. Corbett

**Affiliations:** ^1^Departments of Neurosurgery and Psychiatry, University of Texas Southwestern Medical Center, Dallas, TX, United States; ^2^Department of Biology, Rutgers University Camden, Camden, NJ, United States

**Keywords:** stress, resilience, neuroscience, animal models, molecular mechanisms, neural network mechanisms

A wide range of biological mechanisms induced by stress contribute to the onset and development of disorders like anxiety and depression. However, similar stressors can cause maladaptive changes in behavior in some individuals, but not others. Individuals displaying maladaptive changes in mood or behavior are vulnerable to the adverse effects of stress. Resilient individuals experience a similar stressor but behave as if they were not stressed. The factors contributing to vulnerability or resilience are not completely understood. Vulnerable individuals might be more prone to the stress-induced biological mechanisms that cause a change in mood or behavior. Alternatively, resilient individuals might be able to activate biological mechanisms that prevent changes in mood or behavior that would otherwise be caused by stress. This Research Topic focuses on the subcellular mechanisms that contribute to resilience or vulnerability.

Zhu et al. report that chronic social defeat stress increases glycogen accumulation in astrocytes in the medial prefrontal cortex (mPFC) of mice. The expression levels of seven enzymes important for regulating glycogenesis or glycogenolysis were screened. Of these, brain-type glycogen phosphorylase (PYGB), involved in glycogenolysis, was reduced by stress. Overexpression of PYGB partially ameliorated behavioral assessments of social anxiety and depression, whereas PYGB knockdown exacerbated the adverse effects of stress on social anxiety- and depression-like behavior. These behavioral changes occurred despite relatively modest changes in overall glycogen levels. Together, these findings highlight the importance of PYGB in the mPFC as an important factor contributing to maladaptive behaviors caused by stress.

Gorman-Sandler et al. review the role of mitochondrial dysfunction in stress-related disorders, with a focus on postpartum depression. Women are faced with a wide range of social, psychological, and biological challenges during the postpartum period, which can cause profound effects on stress vulnerability. The importance of this “critical window for vulnerability” is further underscored by stress-related disorders being twice as prevalent in women compared to men. Gorman-Sandler et al. provide a comprehensive review of the biological mechanisms regulated by mitochondria that are disturbed during the postpartum period and may contribute to vulnerability. The biological mechanisms include those involving neural function, hormones, and immune signaling. This review serves as an important resource for demonstrating that mitochondrial dysfunction contributes to affective changes during the postpartum period.

Kigar et al. review how the exposure of rodents to a natural predator causes changes in behavior and specific neural substrates. In these paradigms, rodents are placed in a protective cage within the cage of a freely moving ferret. The cage allows for the exchange of visual, auditory, and olfactory cues without physical contact. Predator stressors are interesting as they are ethologically relevant and purely psychological. These stressors elicit robust behavioral and neuroendocrine changes that are accompanied by alterations in neurotransmitter signaling in stress-related brain regions. Of particular relevance to this topic, ~10% of certain rodent populations exposed to predator stress display a resilient phenotype. Together, Kigar et al. highlight predator stress as an important paradigm for studying the effects of stress on rodent behavior.

The goal of this Research Topic is to better understand the wide range of biological factors contributing to stress resilience or vulnerability. The effects of acute and/or chronic stress can be wide-reaching and affect many molecular mechanisms, brain regions, and biological systems. Understanding the factors contributing to resilience or vulnerability helps us focus on the processes most adversely affected by stress while providing insights into the role of these processes in normal neural function.

## Author contributions

FH: Writing – review & editing. BC: Writing – original draft, Writing – review & editing.

